# Combining Multiple Magnetic Resonance Imaging Sequences Provides Independent Reproducible Radiomics Features

**DOI:** 10.1038/s41598-018-37984-8

**Published:** 2019-02-14

**Authors:** A. Lecler, L. Duron, D. Balvay, J. Savatovsky, O. Bergès, M. Zmuda, E. Farah, O. Galatoire, A. Bouchouicha, L. S. Fournier

**Affiliations:** 1 Department of Neuroradiology, Fondation Ophtalmologique Adolphe de Rothschild, Paris, France; 2 Université Paris Descartes Sorbonne Paris Cité, INSERM UMR-S970, Cardiovascular Research Center - PARCC, Paris, France; 3 Department of Orbitopalpebral Surgery, Fondation Ophtalmologique Adolphe de Rothschild, Paris, France; 4 Sorbonne Paris Cité University, Paris Université Paris Descartes Sorbonne Paris Cité, Assistance Publique-Hôpitaux de Paris, Hôpital Européen Georges Pompidou, Radiology Department, Paris, France

## Abstract

To evaluate the relative contribution of different Magnetic Resonance Imaging (MRI) sequences for the extraction of radiomics features in a cohort of patients with lacrimal gland tumors. This prospective study was approved by the Institutional Review Board and signed informed consent was obtained from all participants. From December 2015 to April 2017, 37 patients with lacrimal gland lesions underwent MRI before surgery, including axial T1-WI, axial Diffusion-WI, coronal DIXON-T2-WI and coronal post-contrast DIXON-T1-WI. Two readers manually delineated both lacrimal glands to assess inter-observer reproducibility, and one reader performed two successive delineations to assess intra-observer reproducibility. Radiomics features were extracted using an in-house software to calculate 85 features per region-of-interest (510 features/patient). Reproducible features were defined as features presenting both an intra-class correlation coefficient ≥0.8 and a concordance correlation coefficient ≥0.9 across combinations of the three delineations. Among these features, the ones yielding redundant information were identified as clusters using hierarchical clustering based on the Spearman correlation coefficient. All the MR sequences provided reproducible radiomics features (range 14(16%)−37(44%)) and non-redundant clusters (range 5–14). The highest numbers of features and clusters were provided by the water and in-phase DIXON T2-WI and water and in-phase post-contrast DIXON T1-WI (37, 26, 26 and 26 features and 14,12, 9 and 11 clusters, respectively). A total of 145 reproducible features grouped into 51 independent clusters was provided by pooling all the MR sequences. All MRI sequences provided reproducible radiomics features yielding independent information which could potentially serve as biomarkers.

## Introduction

Medical imaging is progressively shifting from conventional visual image analysis to quantitative personalized medicine thanks to the recent development of data-driven analysis methods like radiomics^1^. Radiomics is a recently developed field of data-driven research allowing high-throughput mining of vast arrays of quantitative imaging features obtained from routine medical imaging such as CT, PET-CT or MRI. It enables data within digital images to be extracted and analyzed for clinical or research purposes^1,2^. It has shown potential to improve the diagnosis, characterization, therapeutic management and follow-up of many diseases in different organs most particularly in tumors and is evolving rapidly as a potential management tool^2–6^.

The potential of radiomics-based phenotyping in precision medicine is unmatched^7–9^, but the complexity of establishing the radiomics pipeline coupled with the absence of widespread standards can result in a high variability of possible approaches in addition to the risk of generating non-replicable results. One main issue of radiomics data analysis is the risk of overfitting due to the high number of extracted features compared to the comparatively smaller number of patients. That is why one of the first steps of radiomics data analysis consists of data dimensionality reduction to reduce the imbalance between the number of parameters and the size of the dataset and to allow more robust and reliable future statistical analyses. One strategy is to perform a feature selection based on certain technical qualities of features such as reproducibility across different settings, or across readers. There is currently no consensus on the best way to perform data dimensionality reduction, although the majority agrees that there is a global need for validating the radiomics techniques, including the assessment of repeatability, reproducibility, robustness or accuracy to provide strong biomarkers, as recommended by the Imaging Biomarkers Alliances^7–12^. Another stumbling block surrounding the radiomics pipeline is integrating multi-parametric data^7^. Many studies have focused on the analysis of multi-parametric data provided by PET-CT scans, but to the best of our knowledge, no one has yet evaluated the relative contribution of each MR sequence when performing a radiomics analysis, nor does the literature indicate whether there was an added value of using multiple MR sequences vs. only one^5,7,11,13–16^.

The aim of our study was to evaluate the relative contribution of different MRI sequences for the extraction of radiomics features in a cohort of patients with lacrimal gland tumors. We also explored the interactions between features extracted from different MR sequences and proposed a pipeline to rationally reduce feature dimensionality.

## Results

### Demographic and Clinical Characteristics

Thirty-seven patients were included in the study (11 males and 26 females, median age 50 years, IQR [35–59]) with a total of 52 lacrimal gland lesions. Eight patients had a histologically-confirmed orbital lacrimal gland malignant tumor, including 8 lymphomas (2 bilateral) and 2 carcinomas, whereas 29 patients had a benign tumor, including 38 inflammatory lesions (13 bilateral) and 4 pleomorphic adenomas. Twenty-two contralateral normal lacrymal glands were also included. The mean number of voxels per Region Of Interest (ROI) was 880 (range 215–3756).

### Feature reduction according to intra- and inter-observer reproducibility

**Figure 1:**
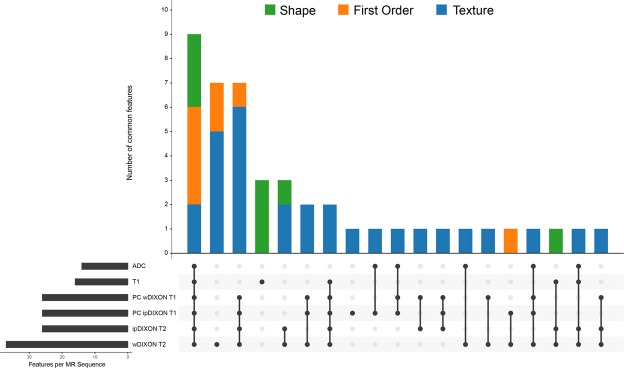
Number of reproducible features extracted according to MR sequences. The number of common features (y-axis) among sequences are displayed in the upper part of the figure with a color-code for each feature category (shape features in green, first-order features in orange and texture features in blue). The lower part of the figure shows the number of sequences sharing the same common features (x-axis). Each black point represents a specific sequence, and the sequences sharing the same common features are linked by a vertical black line. Some features are only displayed by one sequence (only one black point). Apparent Diffusion Coefficient map (ADC); Post-contrast in-phase DIXON T1 (PC ipDIXON T1); Post-contrast water DIXON T1 (PC wDIXON T1); In-phase DIXON T2 (ipDIXON T2); Water DIXON T2 (wDIXON T2).

All the MR sequences provided reproducible radiomics features. The highest number of reproducible features was provided by the wDIXON T2-WI (44%), the ipDIXON T2-WI (31%), the post-contrast wDIXON T1-WI (31%), and the post-contrast ipDIXON T1-WI (31%). The smallest number of reproducible features was provided by the T1-WI (19%) and the ADC map (16%). In total, 45 features (53%) were deemed reproducible in at least one MR sequence, including 7 shape features, 9 first-order features and 28 texture features. Nine features (11%) were reproducible across all MR sequences, including 3 shape features (Area, Convex Area, Minor Axis), 5 first-order features (Number of voxels, Mean, Median, Joint Energy, Root Mean Square (RMS)) and 1 texture feature (GLSZM Zone Variance (ZV)). Two additional texture features were reproducible on all sequences except the ADC map (GLRLM Long Run Emphasis (LRE), GLSZM Large Area Emphasis (LAE)). All these results are detailed in Fig. 1 and Supplementary Figure 1.

### Feature reduction according to feature redundancy

All MR sequences provided multiple clusters of reproducible features. The highest number of clusters (i.e. number of independent information) was provided by the wDIXON T2-WI (n = 14), the ipDIXON T2-WI (n = 12), the post-contrast ipDIXON T1-WI (n = 11) and the post-contrast wDIXON T1-WI (n = 9); the least by the T1-WI (n = 6) and the ADC map (n = 5). The dendrograms of feature clustering are available in Supplementary Figure 2.

### Redundancy of reproducible features and clusters across MR sequences

At step one, 145 reproducible features were shared throughout several sequences. After clustering, we obtained 51 distinct clusters. Features originating from T1-WI and ADC map were never found in the same cluster, as those originating from DIXON-T2-WI and post-contrast DIXON-T1-WI, thereby yielding completely non-redundant information. IpDIXON T2-WI and wDIXON T2-WI shared all their shape features and 5 first-order features (Number of voxels, Joint Energy, Mean, Median, RMS). Post-contrast ipDIXON T1-WI and post-contrast wDIXON T1-WI shared all their shape features and the same 5 first-order features (Number of voxels, Joint Energy, Mean, Median, RMS) as well as 3 texture features (GLRLM Run Length Non-Uniformity (RLN), GLSZM Zone Variance (ZV), GLSZM Zone Size Non-Uniformity (ZSN). The same feature calculated on two sequences derived from the same DIXON acquisitions (in phase and water sequences of both T2 and post-contrast) yielded independent information. We did not find a single sequence that could encompass all the data obtained by one or several other sequences. The clusters obtained from pooled sequences are illustrated in Supplementary Figure 3.

### Effect of ICC and CCC threshold choice on the selection of reproducible features

**Figure 2:**
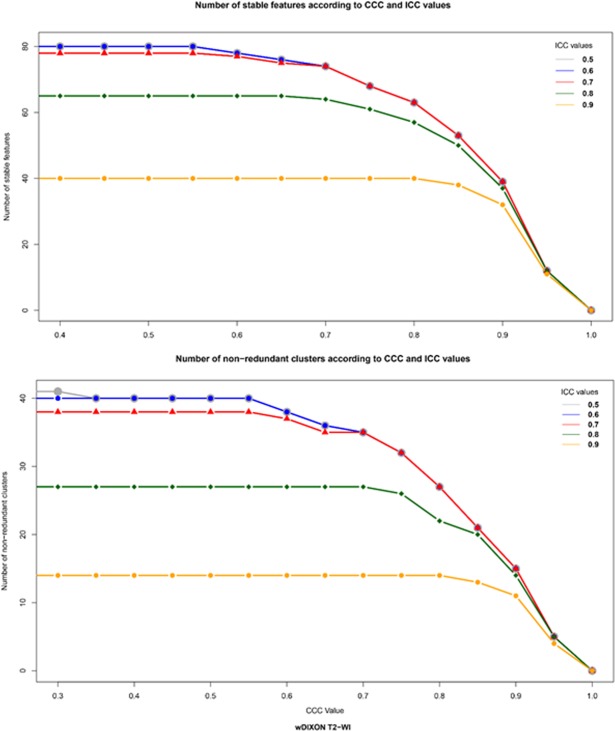
Effect of tuning threshold values of CCC and ICC on the number of reproducible features (**a**) and of non-redundant clusters (**b**). The number of stable features (**a**) or non-redundant clusters (**b**) is displayed in y-axis and the increasing CCC values in x-axis. The colored curves represent distinct ICC values (grey for ICC of 0.5, blue for 0.6, red for 0.7, green for 0.8 and yellow for 0.9). Example on the Water DIXON T2 (wDIXON T2) WI sequence. Intra-class Correlation Coefficient (ICC); Concordance Correlation Coefficient (CCC).

Decreasing ICC and/or CCC thresholds systematically led to an increase in the number of selected features and in non-redundant clusters. However, these changes were essentially observed when the CCC threshold value was < 0.9, whereas no significant difference in terms of number of reproducible features or number of final clusters was observed when modifying the ICC threshold values with a constant CCC threshold value set at 0.9. Results of this threshold tuning using the post-contrast wDIXON T2-WI are plotted in Fig. 2.

## Discussion

Our study showed that all MRI sequences could provide reproducible radiomics features and non-redundant clusters of features and that the different MRI sequences provided additional independent information.

Stability of radiomics features have been extensively studied on CT and PET-CT images^3,7,8,17–19^. However, only a few studies have explored the robustness of these features on MR images^5,13–16^. Image analysis and computer vision of MR images are more complex to develop because of the lack of standardization of MR data, resulting in the absence of tissue specific signal intensities^6^. Despite all these difficulties, several studies have shown promising results from radiomics applied to MR images^5,13–15^. However, these studies only considered one imaging sequence as a source of radiomics features, when clinical use requires the combination of multiple sequences to reach a diagnosis^20,21^. We studied whether considering multiple sequences might enrich the features extracted from MRI data in a radiomics analysis. We focused on the initial step of data dimensionality reduction usually performed in radiomics analyses^6^. We believe that technical validation of radiomics for MRI is of particular importance because there is a substantially higher variability and heterogeneity in images when using MRI as compared to CT or PET-CT. Nevertheless, MRI cannot be replaced by these other imaging modalities in a number of applications in clinical diagnosis.

We showed that each MRI sequence of our protocol could provide reproducible radiomics features and non-redundant clusters. The highest number of features and clusters were provided by the DIXON T2-WI and the post-contrast DIXON T1-WI, which are often the most informative and valuable sequences for characterizing a tumor in a conventional visual approach^22^. Interestingly, the clustering of most reproducible features led to coherent clusters regarding the categories of the features, i.e. geometric, first-order grey-level statistics or texture features. Moreover, although nine features were reproducible on all the sequences, we showed that most of them provided independent information since they were not in the same clusters. This finding advocates for the use of multiparametric MRI protocols when performing radiomics since they provide different and complementary information.

There is no recommendation yet regarding optimal thresholds to use for ICC and CCC when assessing inter- and intra-observer reproducibility. However, authors are often strict and values of 0.8 for ICC and 0.85 to 0.9 for CCC are most often used in the literature^6,8,18,23^. We showed that decreasing the ICC and CCC threshold values led to a higher number of selected features, which is consistent with previous studies^8^. We also showed that the extra features obtained when lowering the thresholds were non-redundant and could not be integrated into already identified clusters. Interestingly, with a constant CCC threshold value of 0.9, modifying the ICC threshold value did not lead to significant differences of features and clusters. It is possible that an overly strict thresholding approach might lead to a loss of valuable information which could be relevant or even crucial to characterizing lesions.

Our study suffers from several limitations. Firstly, as a technical validation study, we assessed the reproducibility and non-redundancy of radiomics-derived MRI features, but we deliberately did not validate their clinical interest. Thus, we do not know whether the extracted features might have any clinical value. We focused on the validation steps of the radiomics technique using MRI rather than evaluating its performance in a single organ. We also believe that strong clinical results cannot be inferred from a relatively small cohort size like ours with unbalanced proportions of diseases.

Secondly, we delineated both healthy and diseased lacrymal glands, with possibly differing proportions of healthy and diseased between observers’ regions of interests, which could have led to a potential sampling bias. We made this choice for two reasons: firstly, we aimed to limit inter and intra-reader segmentation variability by delineating the more visible whole lacrimal gland because a large part of our dataset contained infiltrative disorders like inflammatory or lymphoid disorders with faint borders that make accurate delineation difficult. Second, as the aim of our study was not to answer a clinical question but rather to evaluate whether combining multiple MRI sequences could provide independent reproducible radiomics features, we sampled both healthy and diseased lacrymal glands to extract all the potential reproducible features which could be encountered in the lacrymal gland region. Nevertheless, when pooling sequences, we found that even DIXON sequences provided different reproducible and non-redundant features, whereas the delineation was the same between the in-phase and water contrast sequences derived from the same acquisition. This suggests that these results were independent from the sampling method.

Thirdly, we performed two-dimensional manual delineations only, although a three-dimensional approach and semi-automatic segmentation method might better extract data from the whole tumor environment as well as reduce inter and intra-observer variability^3,17,18,24^. However, despite this limitation, we found a substantial number of reproducible and non-redundant features. We intentionally chose to have one experienced reader specialized in orbital imaging and another one with little experience because future radiomics applications might be performed in non-specialized centers.

Fourthly, it has been shown in the literature that different reconstruction parameters and acquisition modes affect quantitative imaging features^21^. In our single-center prospective study it was not an issue since all the parameters, such as voxel dimension, were consistent throughout the population. However, our results may not be generalizable to all sequences and reconstruction parameters. We selected widely used sequences to reduce this limitation, but further studies in other centers are needed to reinforce the validity of our results.

Next, we explored the reproducibility of MR radiomics features against segmentation variability, but we did not assess their precision against test-rests experiments (repeatability) or across multiple centers and multiple MR devices (reproducibility). As feature reproducibility was reported to be tumor-site specific in a CT study, it would be of great interest to perform and compare studies in different body parts using MRI^8^.

Despite these limitations, our study complied with recommendations, with a high Radiomics Quality Score (RQS) and thus should offer reliable and reproducible data^7^.

In conclusion, we showed that all MRI sequences may provide reproducible radiomics features and non-redundant clusters of features. These findings suggest that future radiomics studies should include several MR sequences in order to increase the probability of finding clinically relevant quantitative biomarkers.

## Methods

### Study Design and Datasets

We conducted a prospective study in a tertiary referral center specializing in ophthalmic diseases. (NCT “02401906”). This study was approved by an independent National Research Ethics Board and adhered to the tenants of the Declaration of Helsinki (IRB “2015-A00364-45”).

From December 2015 to April 2017, 37 patients were included in the study and signed an informed consent prior to inclusion. Inclusion criteria of the prospective study were: (a) age over 18 years; (b) presence of a lacrimal gland lesion; (c) histopathological final diagnosis based on a biopsy or surgery of the mass.

### MR Imaging Acquisition

All MRI exams were performed on the same 3 Tesla Philips INGENIA device with a 32-channel head coil (Philips Medical Systems, Best, Netherlands). MRI protocol was a standard protocol used in clinical practice to explore lacrimal gland lesions including axial T1-WI, axial DWI, coronal T2 DIXON-WI and coronal post-contrast T1 DIXON-WI after administration of intravenous contrast injection of a single bolus (0.1 mmol/kg) of Gadobutrol (Gadovist; Bayer HealthCare, Berlin, Germany). DIXON sequences provide two main contrasts, the first one an in-phase contrast (ipDIXON), the second one a water contrast (wDIXON) where the signal of the fat is not displayed. Acquisition parameters are detailed in Table 1. Apparent Diffusion Coefficient maps (ADC) were calculated voxelwise as the linear slope of signal decrease between b0 and b1000 DWI acquisitions. Patients were asked to look at a fixed point during the acquisitions to prevent kinetic artifacts generated by eye movements.

**Table 1:** MRI acquisition protocol.

	T1-WI	DWI (b0-b1000)	ipDIXON-T2-WI and wDIXON-T2-WI	Post-contrast ipDIXON-T1-WI and wDIXON-T1-WI
Plane	Axial	Axial	Coronal	Coronal
Number of Slices	20	15	30	15
Slice thickness (mm)	2.5, no gap	3, no gap	2, no gap	3, no gap
TR (ms)	622	5750	3000	456
TE (ms)	7	79	80	12
Number of excitations	1	3	2	1
Bandwidth (Hz)	280	1048	260	376
Matrix	480 × 480	176 × 176	448 × 448	320 × 320
FOV (mm)	180 × 180	140 × 140	150 × 150	235 × 235
Acquisition duration (s)	80	184	246	235

WI: Weighted Images; DWI: Diffusion Weighted Imaging; ip: In-Phase; w- Water; TR: Repetition Time; TE: Echo Time; FOV: Field of view; s: seconds.

### Image Analysis and Manual Delineation

Two readers (a senior neuro-radiologist specialized in orbital imaging with 8 years of experience (A.L.) and a junior radiologist with 6 months of experience (L.D.)) were blinded to patient ID, medical history lab results and pathological results. All the post-acquisition steps were performed using an in-house software (Matlab R2013b [The Mathworks, Natick, MA, USA]) adapted from the Vallieres radiomics toolbox^25^.

Each reader performed independently a 2D manual delineation of both lacrymal glands in their entirety for all patients, including healthy tissue as well as the lesion when there was one, and the whole healthy lacrimal gland otherwise. Delineations were performed on each of the MR protocol sequences, resulting in a total of 444 ROIs per reading (2 lacrimal glands per patient on 6 MR sequences for 37 patients). The slice of delineation was independently chosen by each reader, considered as the slice where the lesion had the largest diameter and as the slice where the healthy lacrymal gland had the largest diameter in glands without any lesion. These segmentations were identified as L1 for the first reader and L2.1 for the second reader. A second segmentation session was performed three weeks later by the second reader, identified as L2.2.

### Feature Extraction

All images were pre-processed using an absolute discretization of grey levels using a constant bin width of 20, based on the IBSI recommendations^26^. Neither pixel aggregation nor filtering of the images was performed. Radiomics features were computed allowing the extraction of 85 features per ROI. Thirteen of them were shape features describing geometrical characteristics, 15 were first-order grey-level statistics features describing the intensity and signal distribution, and 57 were texture features describing the spatial distribution of pixel intensities. The texture features were derived from the grey-level co-occurrence matrix (GLCM, 26 features) obtained using 4 angles, grey-level run-length matrix (GLRLM, 13 features), grey-level size-zone matrix (GLSZM, 13 features) and neighbourhood grey-tone difference matrix (NGTDM, 5 features), as described by Zwanenburg *et al*.^1,25–29^. A comprehensive list of features is displayed in Table 2. A total of 37,740 feature values were extracted from each reading (85 features on 444 ROIs).

**Table 2:** Radiomics features extracted using our in-house software.

Shape features (n = 13)
Convex Area Deficit Eccentricity Elongation Extension Major Axis Length Maximum Diameter (MaxDiam) Maximum Geodesic Diameter (MaxDiamGeo) Minor Axis Length Perimeter Perimeter Solidity (Eccentricity) Solidity Surface Area
**First-Order features (n = 15)**
Energy Entropy Interquartile Range 10–90 (Inter1090) Kurtosis Maximum Mean Mean Absolute Deviation (MAD) Median Minimum Number of Pixels (Npix) Range Root Mean Square (RMS) Skewness Standard Deviation (STD) Uniformity
**Grey-Level Co-occurrence Matrix (GLCM) (n = 26)**
Agreement Autocorrelation Cluster Prominence Cluster Shade Cluster Tendency Contrast Correlation 1 Correlation 2 Difference Entropy Dissimilarity Homogeneity 1 Homogeneity 2 Informal Measure of Correlation1 (IMC1) Informal Measure of Correlation2 (IMC2) Inverse Difference Moment (IDM) Inverse Difference Moment Normalized (IDMN) Inverse Variance Joint Energy Joint Entropy Maximum Probability Sum Average Sum Entropy Sum Mean Sum Variance Variance 1 Variance 2
**Grey-Level Size Zone Matrices (GLSZM) (n = 13)**
Grey Level Non-Uniformity (GLN) Grey Level Variance (GLV) High Grey Level Zone Emphasis (HGLZE) Large Area Emphasis (LAE) Large Area High Grey Level Emphasis (LAHGLE) Large Area Low Grey Level Emphasis (LALGLE) Low Grey Level Zone Emphasis (LGLZE) Size-Zone Non-Uniformity (ZSN) Small Area Emphasis (SAE) Small Area High Grey Level Emphasis (SAHGLE) Small Area Low Grey Level Emphasis (SALGLE) Zone Percentage (ZP) Zone Variance (ZV)
**Grey-Level Run Length Matrices (GLRLM) (n = 13)**
Grey Level Variance (GLV) Grey-Level Non-Uniformity (GLN) High Grey Level Run Emphasis Long Run Emphasis (LRE) Long Run High Grey Level Emphasis Long Run Low Grey Level Emphasis Low Grey Level Run Emphasis (LGLRE) Run Length Non-Uniformity (RLN) Run Percentage (RP) Run Variance (RV) Short Run Emphasis (SRE) Short Run High Grey Level Emphasis Short Run Low Grey Level Emphasis
**Neighbouring Grey Tone Difference Matrices (NGTDM) (n = 5)**
Busyness Coarseness Complexity Contrast Strength

Mathematical definitions are detailed in the Imaging Biomarker Standardization Initiative (IBSI) guidelines and the Matlab toolbox documentation^25,28^.

### Feature reduction according to intra- and inter-observer reproducibility

The first step for data dimensionality reduction was done using a 2-way mixed intra-class correlation coefficient (ICC) (absolute agreement, average type) on the three paired combinations of readings (L1/L2.1, L1/L2.2, L2.1/L2.2) and the Lin’s concordance correlation coefficient (CCC) on the pair (L2.1/L2.2) to assess the inter and intra-observer feature stability^30,31^. A feature was defined as highly reproducible if all three ICC values were ≥ 0.8 and the CCC value was ≥ 0.9.

### Feature reduction according to feature redundancy

In a second step, we performed hierarchical clustering of the previously selected reproducible features using the Spearman correlation coefficient as the distance criterion. As many reproducible features obtained at step one were susceptible to being shared by several sequences, we explored if these shared features gave the same or independent information. Features presenting a Spearman correlation coefficient value above 0.9 were considered redundant and were grouped in the same cluster. This action provided statistically independent information by clustering what was redundant. We first performed the clustering on each MR sequence independently. We also clustered pooled reproducible features obtained from each MR sequence to evaluate whether sequences yielded the same reproducible and non-redundant features, or if they contributed complementary information.

The stability and clustering steps were also computed with various ICC and CCC threshold values to explore the impact of modifying threshold values on the number of robust features and on the number of feature clusters. Particularly, we explored if decreasing the ICC and CCC thresholds led to a higher number of clusters or if only additional redundant features were appended. All statistical analyses were performed using R-3.3.3 (R Foundation, Vienna, Austria)^32^.

Evaluation criteria and reporting guidelines have been recently published to improve reliability, comparability and generalizability of radiomics-based studies^7,8^. Our study was compliant with most of the checkpoints a technical validation study must have: we performed a prospective study with a standardized and carefully chosen imaging protocol for all patients; we tested the reproducibility of radiomics features against multiple segmentations using manual segmentation and simultaneously assessed intra and inter-observer variability; we used meaningful selection methods to perform data dimensionality reduction and to avoid potential overfitting; we shared all details about our methods to allow independent replication of our results. Finally, we used a cohort of patients with lacrimal gland lesions, a field that is poorly known by the radiological community, which increases the need for finding potential imaging biomarkers to improve the accuracy of non-invasive diagnosis.

### Ethical Approval

1. This study was approved by an independant National institutional Research Ethics Board (IRB “2015-A00364-45”).
2. The study adhered to the tenants of the Declaration of Helsinki.
3. Signed informed consent was obtained from all subjects.

## Supplementary information

Supplementary Figures

## Data Availability

In this article, the images are deidentified MRI data, from MRIs performed in our center for this specific study. Anonymized data not published within the article will be shared following a reasonable request from a qualified investigator.
