# Supplementary material for: Combining Multiple Magnetic Resonance Imaging Sequences Provides Independent Reproducible Radiomics Features
Source: Sci Rep. 2019 Feb 14;9:2068. doi: 10.1038/s41598-018-37984-8 (PMC6376058; doi:10.1038/s41598-018-37984-8)
Supplement: Supplementary file 1 — Supplementary Figures [file 41598_2018_37984_MOESM1_ESM.pdf]

Combining Multiple Magnetic Resonance Imaging Sequences Provides Independent Reproducible Radiomics Features

A.Lecler*, MD, PhD[,2]; L.Duron*, MD, MSc[1,2] ; D.Balvay[2], PhD; J.Savatovsky, MD[1] ; O.Bergès, MD[1] ; M. Zmuda, MD[3]; E.Farah, MD[3] ; O.Galatoire, MD[3] ; A.Bouchouicha, PhD[2] ; L.S.Fournier, MD, PhD[2,4]

*These authors contributed equally to the work

1. Department of Radiology, Fondation Ophtalmologique Adolphe de Rothschild, Paris, France
2. Université Paris Descartes Sorbonne Paris Cité, INSERM UMR-S970, Cardiovascular Research Center - PARCC, Paris, France
3. Department of Orbitopalpebral Surgery, Fondation Ophtalmologique Adolphe de Rothschild, Paris, France
4. Sorbonne Paris Cité University, Paris Université Paris Descartes Sorbonne Paris Cité, Assistance Publique-Hôpitaux de Paris, Hôpital Européen Georges Pompidou, Radiology Department, Paris, France

Corresponding author:

Augustin Lecler, MD

Department of Radiology, Fondation Ophtalmologique A. de Rothschild

29 rue Manin, 75019 Paris, France

E-mail: alecler@for.paris

Phone: +00 33 1 48 03 64 01 Fax : +00 33 1 48 03 65 01

Supplementary Figures

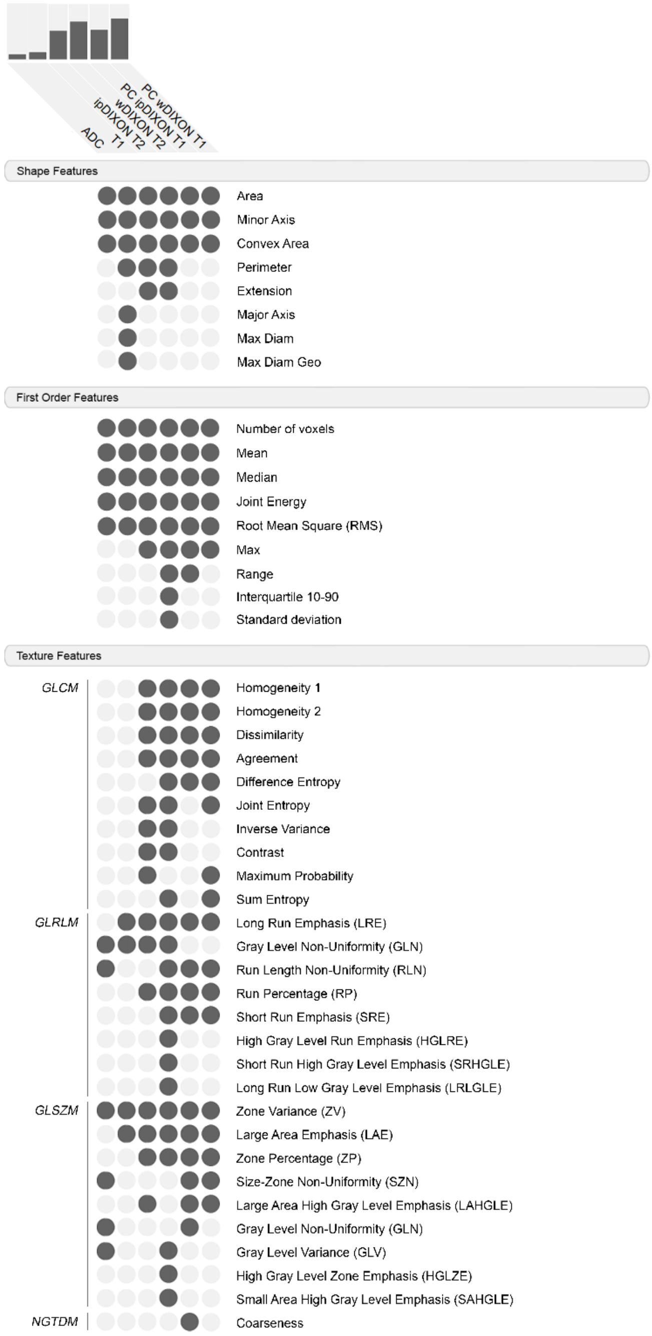

Supplementary Figure 1. Details of reproducible features extracted after the step one. Each MR sequence is displayed in a column. Each row displays a distinct feature. Features issued from the same categories (shape, first-order or texture) are grouped. A black point indicates a reproducible feature. Apparent Diffusion Coefficient map (ADC); Post-contrast in-phase DIXON T1 (PC ipDIXON T1); Post-contrast water DIXON T1 (PC wDIXON T1);

In-phase DIXON T2 (ipDIXON T2); Water DIXON T2 (wDIXON T2); grey-level co-occurrence (GLCM), grey-level run-length (GLRLM), grey-level size-zone (GLSZM), neighborhood grey-tone difference (NGTDM).

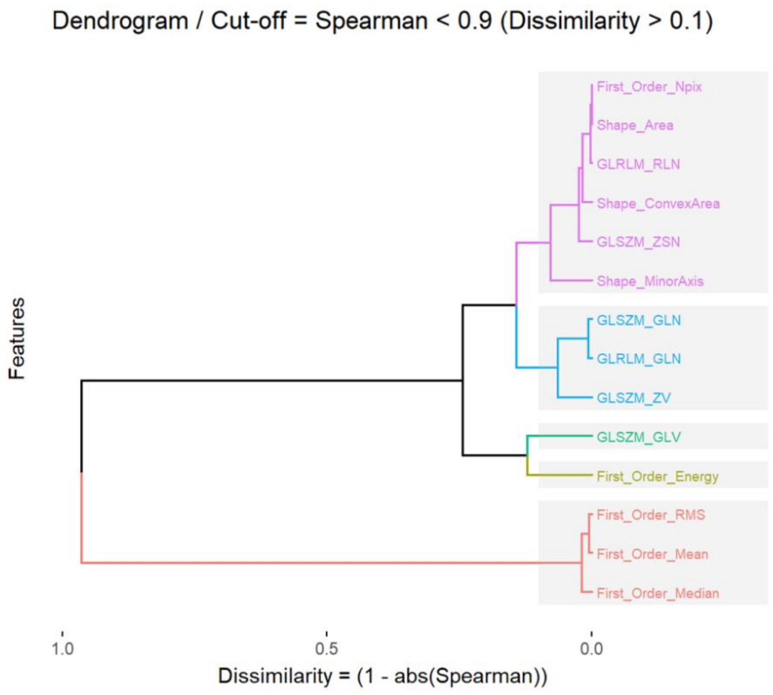
Dendrogram / Cut-off = Spearman < 0.9 (Dissimilarity > 0.1)
First_Order_Npix
Shape_Area
GLRLM_RLN
Shape_ConvexArea
GLSZM_ZSN
Shape_MinorAxis
GLSZM_GLN
GLRLM_GLN
GLSZM_ZV
GLSZM_GLV
First_Order_Energy
First_Order_RMS
First_Order_Mean
First_Order_Median
Features
1.0
0.5
0.0
Dissimilarity = (1 - abs(Spearman))

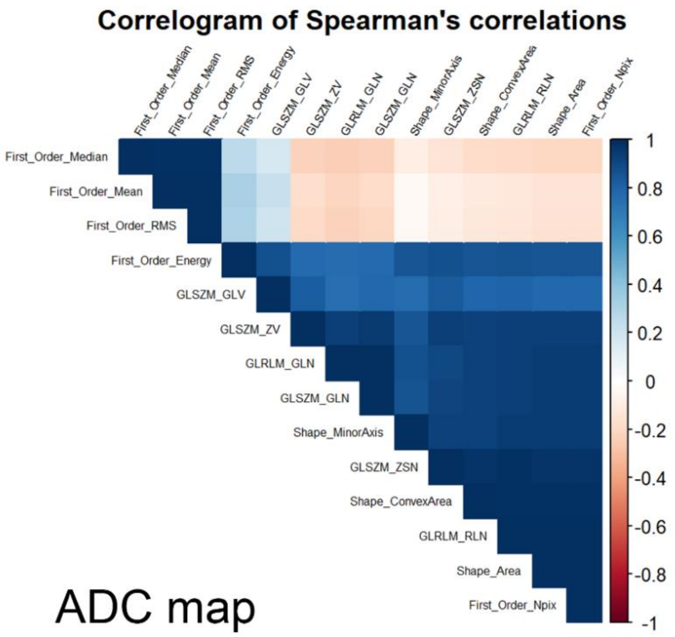
Correlogram of Spearman's correlations
First_Order_Median
First_Order_Mean
First_Order_RMS
First_Order_Energy
GLSZM_GLV
GLSZM_ZV
GLRLM_GLN
GLSZM_GLN
Shape_MinorAxis
GLSZM_ZSN
Shape_ConvexArea
GLRLM_RLN
Shape_Area
First_Order_Npix
1
0.8
0.6
0.4
0.2
0
-0.2
-0.4
-0.6
-0.8
-1
ADC map

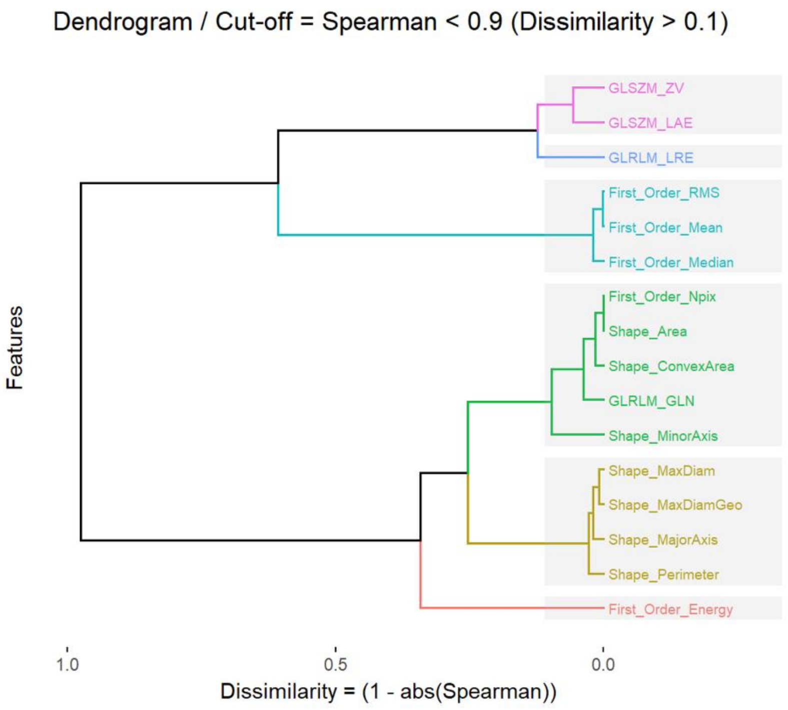
Dendrogram / Cut-off = Spearman < 0.9 (Dissimilarity > 0.1)
Features
GLSZM_ZV
GLSZM_LAE
GLRLM_LRE
First_Order_RMS
First_Order_Mean
First_Order_Median
First_Order_Npix
Shape_Area
Shape_ConvexArea
GLRLM_GLN
Shape_MinorAxis
Shape_MaxDiam
Shape_MaxDiamGeo
Shape_MajorAxis
Shape_Perimeter
First_Order_Energy
1.0
0.5
0.0
Dissimilarity = (1 - abs(Spearman))

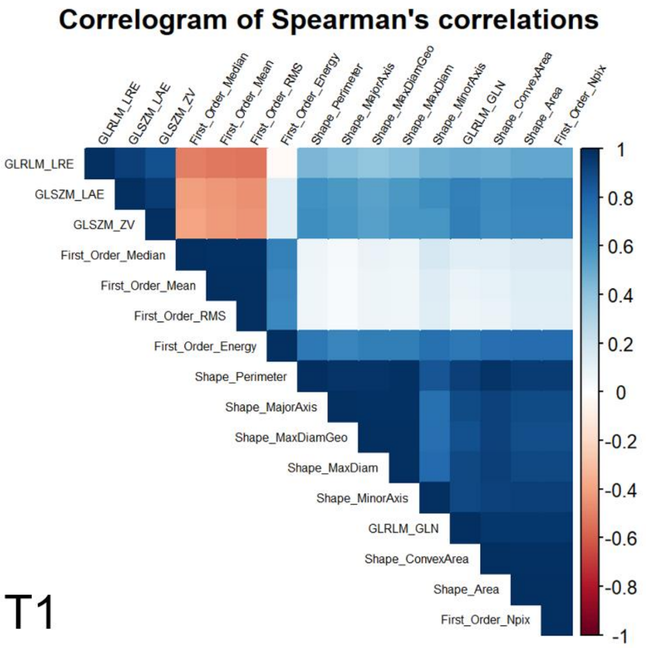
Correlogram of Spearman's correlations
GLRLM_LRE
GLSZM_LAE
GLSZM_ZV
First_Order_Median
First_Order_Mean
First_Order_RMS
First_Order_Energy
Shape_Perimeter
Shape_MajorAxis
Shape_MaxDiamGeo
Shape_MaxDiam
Shape_MinorAxis
GLRLM_GLN
Shape_ConvexArea
Shape_Area
First_Order_Npix
1
0.8
0.6
0.4
0.2
0
-0.2
-0.4
-0.6
-0.8
-1

T1

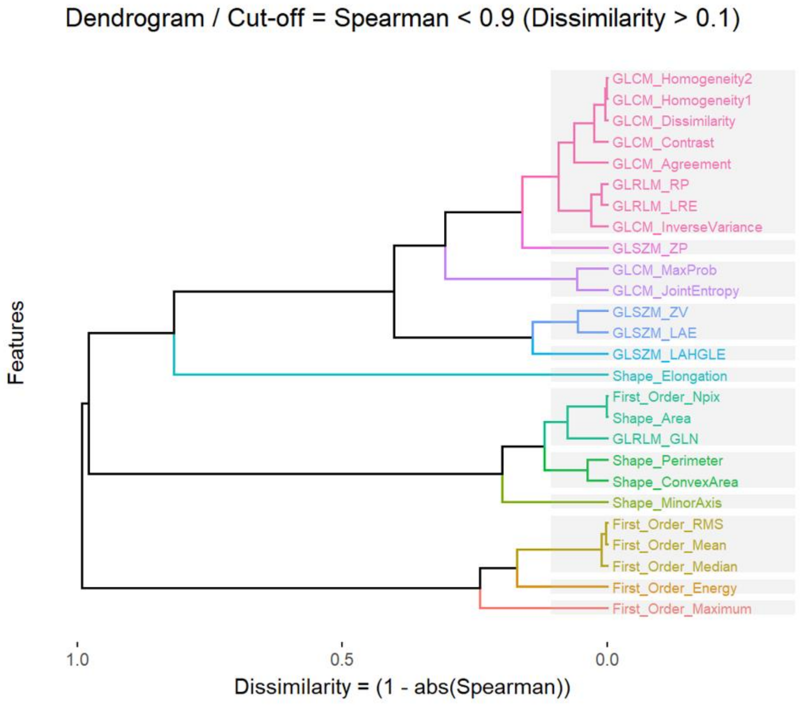
Dendrogram / Cut-off = Spearman < 0.9 (Dissimilarity > 0.1)
Features
GLCM_Homogeneity2
GLCM_Homogeneity1
GLCM_Dissimilarity
GLCM_Contrast
GLCM_Agreement
GLRLM_RP
GLRLM_LRE
GLCM_InverseVariance
GLSZM_ZP
GLCM_MaxProb
GLCM_JointEntropy
GLSZM_ZV
GLSZM_LAE
GLSZM_LAHGLE
Shape_Elongation
First_Order_Npix
Shape_Area
GLRLM_GLN
Shape_Perimeter
Shape_ConvexArea
Shape_MinorAxis
First_Order_RMS
First_Order_Mean
First_Order_Median
First_Order_Energy
First_Order_Maximum
1.0
0.5
0.0
Dissimilarity = (1 - abs(Spearman))

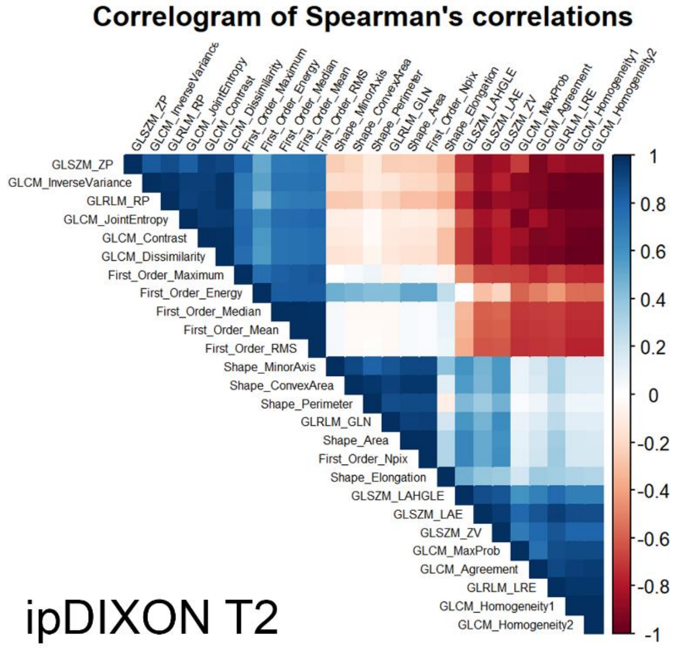
Correlogram of Spearman's correlations
GLSZM_ZP
GLCM_InverseVariance
GLRLM_RP
GLCM_JointEntropy
GLCM_Contrast
GLCM_Dissimilarity
First_Order_Maximum
First_Order_Energy
First_Order_Median
First_Order_Mean
First_Order_RMS
Shape_MinorAxis
Shape_ConvexArea
Shape_Perimeter
GLRLM_GLN
Shape_Area
First_Order_Npix
Shape_Elongation
GLSZM_LAHGLE
GLSZM_LAE
GLSZM_ZV
GLCM_MaxProb
GLCM_Agreement
GLRLM_LRE
GLCM_Homogeneity1
GLCM_Homogeneity2
1
0.8
0.6
0.4
0.2
0
-0.2
-0.4
-0.6
-0.8
-1
ipDIXON T2

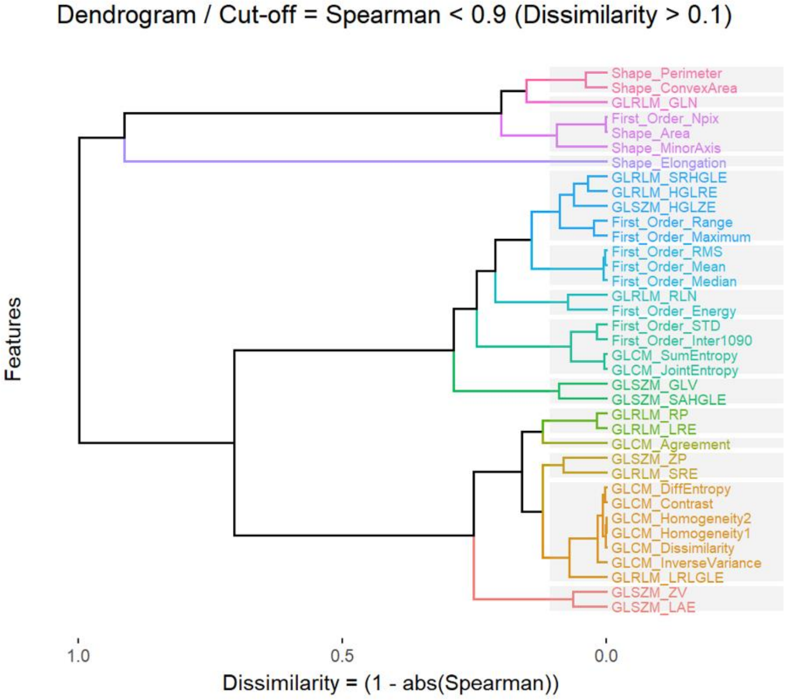
Dendrogram / Cut-off = Spearman < 0.9 (Dissimilarity > 0.1)
Features
Shape_Perimeter
Shape_ConvexArea
GLRLM_GLN
First_Order_Npix
Shape_Area
Shape_MinorAxis
Shape_Elongation
GLRLM_SRHGLE
GLRLM_HGLRE
GLSZM_HGLZE
First_Order_Range
First_Order_Maximum
First_Order_RMS
First_Order_Mean
First_Order_Median
GLRLM_RLN
First_Order_Energy
First_Order_STD
First_Order_Inter1090
GLCM_SumEntropy
GLCM_JointEntropy
GLSZM_GLV
GLSZM_SAHGLE
GLRLM_RP
GLRLM_LRE
GLCM_Agreement
GLSZM_ZP
GLRLM_SRE
GLCM_DiffEntropy
GLCM_Contrast
GLCM_Homogeneity2
GLCM_Homogeneity1
GLCM_Dissimilarity
GLCM_InverseVariance
GLRLM_LRLGLE
GLSZM_ZV
GLSZM_LAE
1.0
0.5
0.0
Dissimilarity = (1 - abs(Spearman))

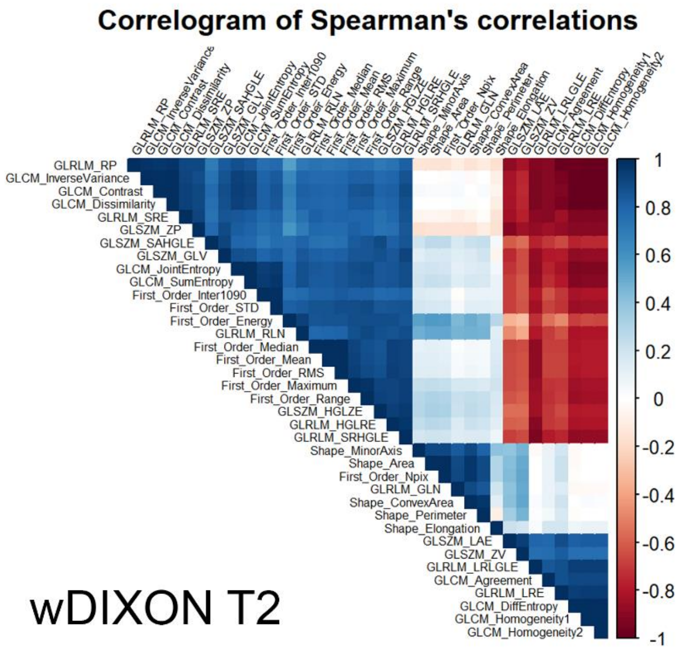
Correlogram of Spearman's correlations
wDIXON T2
1
0.8
0.6
0.4
0.2
0
-0.2
-0.4
-0.6
-0.8
-1

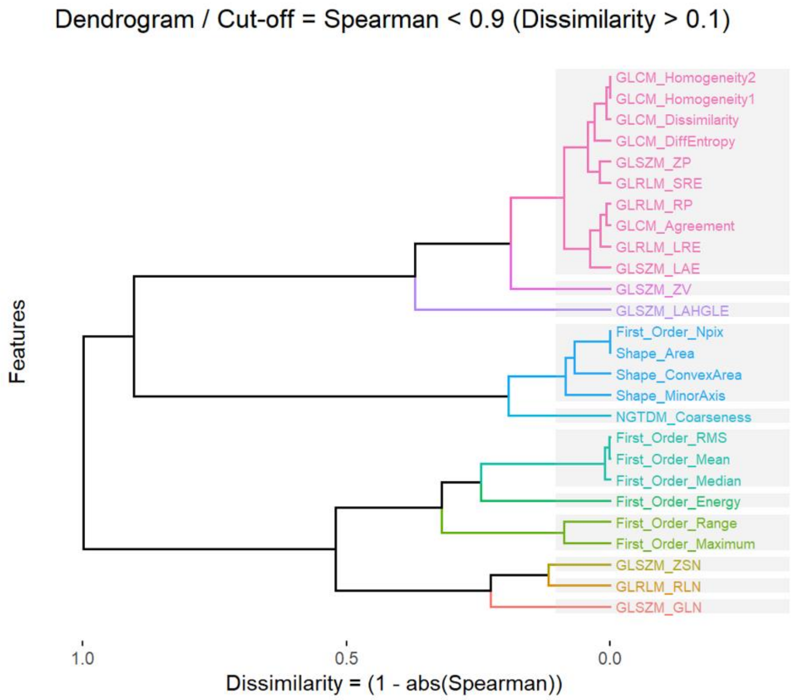
Dendrogram / Cut-off = Spearman < 0.9 (Dissimilarity > 0.1)
Features
GLCM_Homogeneity2
GLCM_Homogeneity1
GLCM_Dissimilarity
GLCM_DiffEntropy
GLSZM_ZP
GLRLM_SRE
GLRLM_RP
GLCM_Agreement
GLRLM_LRE
GLSZM_LAE
GLSZM_ZV
GLSZM_LAHGLE
First_Order_Npix
Shape_Area
Shape_ConvexArea
Shape_MinorAxis
NGTDM_Coarseness
First_Order_RMS
First_Order_Mean
First_Order_Median
First_Order_Energy
First_Order_Range
First_Order_Maximum
GLSZM_ZSN
GLRLM_RLN
GLSZM_GLN
1.0
0.5
0.0
Dissimilarity = (1 - abs(Spearman))

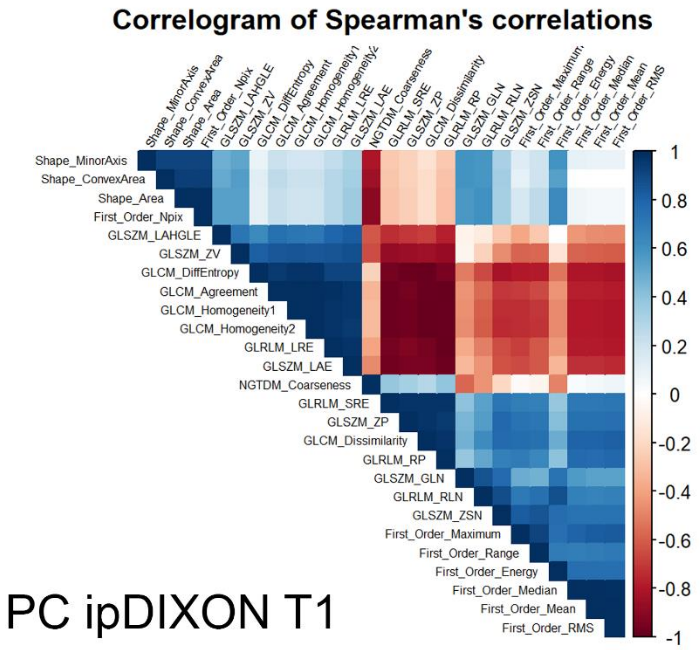
Correlogram of Spearman's correlations
Shape_MinorAxis
Shape_ConvexArea
Shape_Area
First_Order_Npix
GLSZM_LAHGLE
GLSZM_ZV
GLCM_DiffEntropy
GLCM_Agreement
GLCM_Homogeneity1
GLCM_Homogeneity2
GLRLM_LRE
GLSZM_LAE
NGTDM_Coarseness
GLRLM_SRE
GLSZM_ZP
GLCM_Dissimilarity
GLRLM_RP
GLSZM_GLN
GLRLM_RLN
GLSZM_ZSN
First_Order_Maximum
First_Order_Range
First_Order_Energy
First_Order_Median
First_Order_Mean
First_Order_RMS
1
0.8
0.6
0.4
0.2
0
-0.2
-0.4
-0.6
-0.8
-1
PC ipDIXON T1

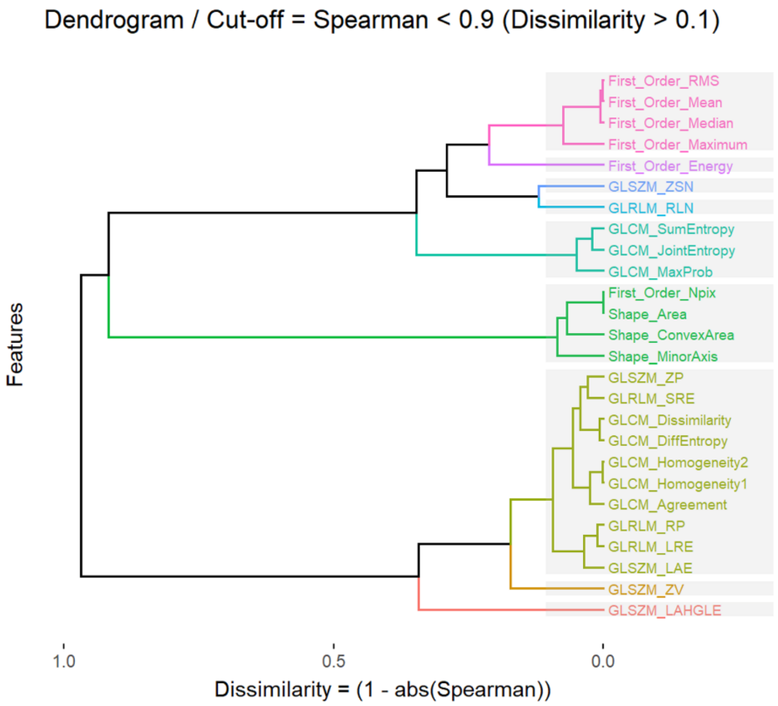
Dendrogram / Cut-off = Spearman < 0.9 (Dissimilarity > 0.1)
Features
First_Order_RMS
First_Order_Mean
First_Order_Median
First_Order_Maximum
First_Order_Energy
GLSZM_ZSN
GLRLM_RLN
GLCM_SumEntropy
GLCM_JointEntropy
GLCM_MaxProb
First_Order_Npix
Shape_Area
Shape_ConvexArea
Shape_MinorAxis
GLSZM_ZP
GLRLM_SRE
GLCM_Dissimilarity
GLCM_DiffEntropy
GLCM_Homogeneity2
GLCM_Homogeneity1
GLCM_Agreement
GLRLM_RP
GLRLM_LRE
GLSZM_LAE
GLSZM_ZV
GLSZM_LAHGLE
1.0
0.5
0.0
Dissimilarity = (1 - abs(Spearman))

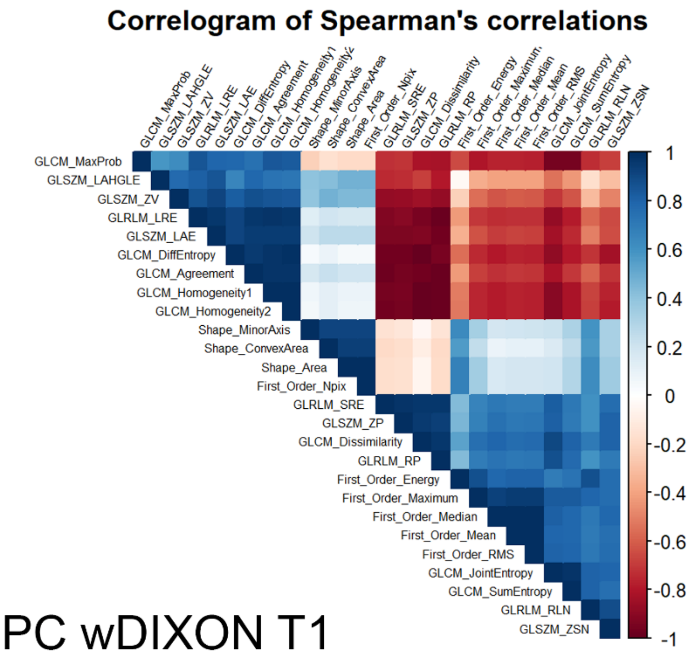
Correlogram of Spearman's correlations
GLCM_MaxProb
GLSZM_LAHGLE
GLSZM_ZV
GLRLM_LRE
GLSZM_LAE
GLCM_DiffEntropy
GLCM_Agreement
GLCM_Homogeneity1
GLCM_Homogeneity2
Shape_MinorAxis
Shape_ConvexArea
Shape_Area
First_Order_Npix
GLRLM_SRE
GLSZM_ZP
GLCM_Dissimilarity
GLRLM_RP
First_Order_Energy
First_Order_Maximum
First_Order_Median
First_Order_Mean
First_Order_RMS
GLCM_JointEntropy
GLCM_SumEntropy
GLRLM_RLN
GLSZM_ZSN
1
0.8
0.6
0.4
0.2
0
-0.2
-0.4
-0.6
-0.8
-1
PC wDIXON T1

Supplementary Figure 2. Dendrograms and Spearman's Correlation Matrices showing the reproducible features and clusters for each MR sequence with the threshold values ICC 0.8 / CCC 0.9. On the dendrograms, the features are displayed in the y-axis, and the dissimilarity in the x-axis (low dissimilarity on the right and high dissimilarity on the left of the axis). Two features or clusters of features are grouped by a branch according to their dissimilarity. All features with a dissimilarity < 0.1 indicating a high redundancy share the same colors and are grouped in the same grey box. All clusters with a dissimilarity ≥ 0.1 are considered independent. On the correlograms, the features are displayed in both x-axis and y-axis. Similarities between features compared two-by-two are color-coded in blue for a positive Spearman's correlation and in red for a negative correlation. The intensity of the correlation is represented by the intensity of the color, with higher correlations coded with darker colors and low correlations with lighter colors.

ADC map (a), T1-WI (b), ipDIXON T2-WI (c), wDIXON T2-WI (d), ipDIXON T1-WI (e), wDIXON T1-WI (f). Grey-level co-occurrence (GLCM), grey-level run-length (GLRLM), grey-level size-zone (GLSZM), neighborhood grey-tone difference.

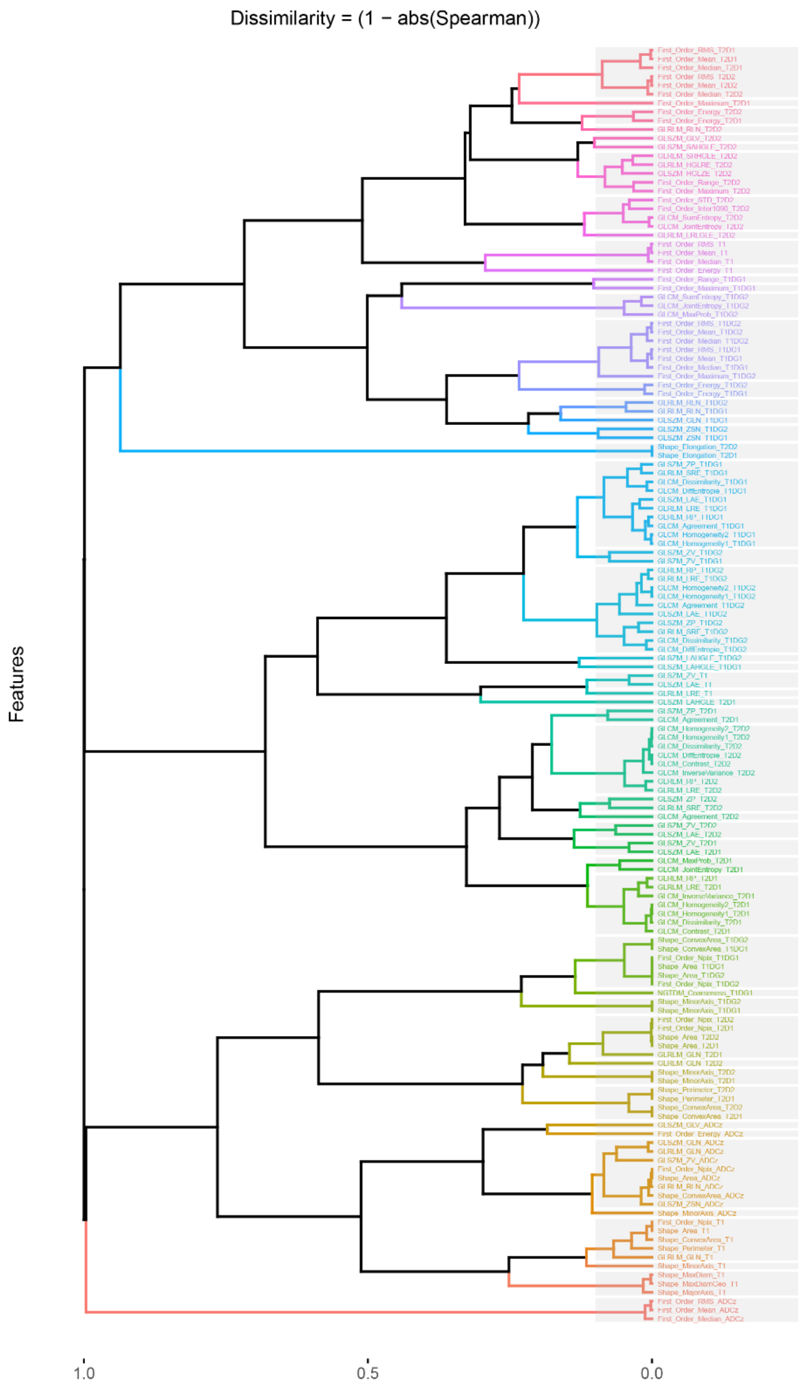

Supplementary Figure 3. Dendrogram showing the reproducible features and clusters of multiple sequences pooled together with the threshold values ICC 0.8 / CCC 0.9. The features are displayed in the y-axis and the dissimilarity in the x-axis (low dissimilarity on the right and high dissimilarity on the left of the axis). Two features or clusters of features are grouped by a branch according to their dissimilarity. All features with a dissimilarity < 0.1 indicating a high redundancy share the same colors and are grouped in the same grey box. All

clusters with a dissimilarity ≥ 0.1 are considered independent. Gray-level co-occurrence (GLCM), gray-level run-length (GLRLM), gray-level size-zone (GLSZM), neighborhood grey-tone difference (NGTDM).
